# Prevalence, correlates, and behavioral outcomes of alcohol gifting in China

**DOI:** 10.1186/s12889-022-13946-8

**Published:** 2022-08-31

**Authors:** Lu Zhang, Lixin Huang, Caitlin Weiger, Can Jiao, Ying Li, Dan Wu

**Affiliations:** 1 School of Psychology/Center for Mental Health, Shenzhen University, Shenzhen, Guangdong 518060 China; 2 Bloomberg School of Public Health, John Hopkins University, Baltimore, 21205 USA; 3 School of Public Health, Xi’an Medical University, Xi’an, Shaanxi 710021 China

**Keywords:** Alcohol gifting, Social capital, Alcohol consumption, Behavioral outcome

## Abstract

**Introduction:**

Alcohol gifting is a very common practice in China. However, little is known about the potentially adverse consequences of alcohol gifting. This study aimed to investigate the prevalence of, and factors associated with, alcohol gifting, and explore whether drinking and tobacco use were associated with alcohol gifting.

**Methods:**

Using a cross-sectional multi-stage survey, a sample of 982 household heads from Guangdong Province and 530 household heads from Shaanxi Province was collected online from 30 April to 30 July 2020 in China. Participants completed questionnaires regarding socio-demographic characteristics, social capital, drinking status, and gifting alcohol behavior. Chi-square analysis and multiple logistic regression analysis were used to identify the factors associated with alcohol gifting, and to identify its relationship with alcohol and cigarette use status.

**Results:**

Multiple logistic regression analysis showed that age, gender, household annual income, province, drinking status, and social participation were prominent correlates of both offering and receiving alcohol. Participants who were married, had an education level of junior high school, or had a large social network had higher odds of receiving alcohol. When both alcohol gifting behaviors were included in the models, participants who offered alcohol had 2.15 (95% CI: 1.63–2.85) times higher odds of current drinking than those who didn’t offer alcohol and participants who received alcohol had 1.87 (95% CI: 1.45–2.41) times higher odds of current drinking than those who did not receive alcohol. Those who received alcohol had significantly higher odds of current smoking (AOR = 1.64; 95% CI: 1.25–2.14), while those who offered alcohol had significantly lower odds of current smoking (AOR = 0.71;95% CI:0.53–0.95).

**Conclusions:**

Social participation is an important correlate of alcohol gifting. Alcohol receiving behaviors were significantly associated with both current alcohol and tobacco use. These associations can be used to inform alcohol gifting interventions in China.

## Background

Alcohol is a psychoactive substance that can produce addiction and dependence [1]. Chronic alcohol use is associated with a myriad of negative health outcomes, including damage to the central nervous system [1]. Alcohol use is causally associated with more than 200 diseases and injuries and is a major contributor to mortality globally [1]. The WHO global status report on alcohol and health reported that 3 million deaths and millions of disabilities are caused by alcohol consumption each year, which constitutes over 5.3% of deaths worldwide [2]. However, drinking behavior remains very common, especially in China. In 2015–2016, the prevalence of alcohol use in China, defined as the percentage of people who have drunk alcohol in the past 12 months, was 43.7% among adults 18 years of age and older. Prevalence is higher among adult men (64.5%) than adult women (23.1%) [3].

Multiple factors are associated with alcohol consumption, including biological [4], sociocultural [5], and psychological factors [6, 7]. For example, genetics appears to play a critical role in alcohol dependence and consumption. Polymorphisms in Alcohol Dehydrogenase Genes, specifically, can lead to an increased risk of alcohol dependence [8–10]. Ecological Systems Theory posits that people’ s behavior is influenced by nested ecological systems, including microsystem, mesosystem, exosystem, and macrosystem. Macrosystems, which refer to as the culture, subculture, and social environment, are of particular interest [11]. The macrosystem in China, where there is a strong historical influence of Confucian culture, may figure prominently in Chinese alcohol use, particularly among men. The Confucian culture emphasizes developing and maintaining social bonds via gift exchange, which is seen as a social norm [5, 12, 13]. Chinese consumers spend more money on alcohol when purchasing alcohol as a gift, compared to when it is purchased for their own use. This may reflect a desire for people to be perceived favorably among their peers [14]. This idea is further supported by findings that gifting alcohol serves as a mechanism to maintain good relationships with elders and promote camaraderie among peers, [15] especially in higher social classes [16]. Gender ideals are also associated with alcohol use, where men who consumed alcohol are seen as full of masculine charm and loyalty [15, 17]. While global per capita alcohol consumption rose from 5.5 L in 2005 to 6.4 L in 2016, per capita alcohol consumption in China rose to an even greater extent, from 4.1 L in 2005 to 7.2 L in 2016. This increasing rate of per capita alcohol consumption may indicate a great challenge for alcohol control in China [1].

Identifying factors associated with alcohol gifting behavior in China may inform future interventions. However, there is little published literature on this topic. One of the few existing studies showed that spending more money on wine gifting in China is associated with younger age and higher education [18]. Top reasons for consuming wine included business, while a top reason for purchasing wine was gifting. It should be noted, however, that these relationships often vary by region [18]. Although this study offers some insight into correlates with demographic factors, the influence of the social environment should also be considered. Social Capital Theory predicts that individuals with strong social capital inherently have access to more supportive resources and have a higher capacity to utilize them [19, 20]. Higher social capital can result in the spread of health information, such as information about the harms of drinking, via social networks, which can influence health-related behaviors [21]. According to Social Capital Theory and Behavioral Accessibility Theory, alcohol gifting as a norm may further increase contact with and consumption of alcohol for both non-drinkers and drinkers attempting to quit.

It is also important to consider the potential adverse consequences of alcohol exchange in addition to factors and characteristics associated with alcohol gifting. Given the hypothesis proposed by previous empirical research [22], gifting alcohol may be associated with alcohol use. However, there is a lack of evidence-based studies that quantitatively identify the relationship between alcohol gifting behavior and potential hazardous behavioral outcomes, such as alcohol drinking and tobacco smoking in China. Although there is no research on this relationship in China, there have been studies assessing this relationship in other countries. Reviews from the U.S. identified a significantly higher risk for alcohol misuse among those who use tobacco [23]. Nationally representative data from the U.S.-based Add Health Survey also found a high prevalence of polysubstance use behavior, including the use of alcohol, marijuana, and cigarettes among adolescents in 2008 [24]. Polysubstance use of alcohol and tobacco is particularly concerning because they enhance the effects of each other, a reaction that tobacco and alcohol companies have exploited to promote sales [23].

The purpose of this study is to investigate the prevalence and correlates of alcohol gifting, including associations with social capital. We additionally aim to explore whether alcohol gifting is associated with alcohol or tobacco consumption in China. We employ quantitative analysis on a large sample at the regional/provincial level to inform evidence-based alcohol control practices relevant to China's alcohol gifting culture.

## Methods

### Study design and participants

A multistage sampling design was utilized in this study and the sample consisted of the heads of households (HHs) from two provinces in China. Guangdong and Shaanxi Province were selected based on their regional diversity and existing research collaboration. Guangdong is a southeastern coastal province with a population of 126.84 million and $14,546. per capita GDP, whereas Shaanxi is a northwestern inland province with 39.54 million people and $11,153 per capita GDP in 2021 [25]. HH refers to the head of the family on the household register. In China, the head of the household is the person in charge of the current household [26]. One university each from Guangdong and Shaanxi Province was selected based on their regional diversity and existing research collaboration with the primary investigators. Within the two universities, all students that had health professional courses were invited to collect data as investigators. A survey link was given to all eligible students, which they were encouraged to distribute to their parents. In total, 982 HHs from Guangdong Province and 530 HHs from Shaanxi Province consented to participate in the study. More detailed information on the sampling and recruitment process can be found in Wu, et al. [22]. The online survey was developed on the Wenjuanxing Platform (https://www.wjx.cn/app/survey.aspx) and conducted from April 30 to July 30, 2020. The study protocol was approved by the Ethics Committee of Guangdong Medical University, and all participants provided written informed consent before they began the survey.

### Measures

#### Socio-demographic characteristics

Socio-demographic information was collected, including age, gender, place of residence, ethnicity, marital status, educational attainment, and per capita annual family income. Given the participants in this survey were parents of college students which are probably between 45–50 years old in general [27], the age category was divided into “ < 45”, “ 45–49”, and “ > 49”.

#### Social capital

Participants’ social capital was assessed using the 12-item Social Capital Questionnaire [28], which has acceptable internal reliability. The higher this score on this scale, the greater the social capital. The Social Capital Questionnaire assesses the three factors of social capital: cognitive social capital, social participation, and social network. We analyze the subscales separately. The sub-scale for cognitive social capital contained four questions, and the Cronbach’s alpha coefficient was 0.786. The sub-scale for social participation contained four questions, and the Cronbach’s alpha coefficient was 0.805, suggesting good reliability. Social network was assessed by ascertaining the number of good friends, trustable classmates, helpful neighbors, close relatives, and cooperative partners. Cronbach’s alpha coefficient for social network was 0.827, which suggests good reliability of this sub-scale.

#### Drinking status

Drinking status was ascertained by asking respondents on how many days they drank during the past month using the following response options: Yes, drank every day; Yes, drank on one or more days, but not every day; no days [29]. Daily drinkers were defined as drinking every day, occasional drinkers were defined as drinking on one or more days, but not every day, and current non-drinkers were defined as those who did not drink in the past month, including never-drinker and former drinker [30, 31]. Categories for occasional drinkers and daily drinkers were combined and compared to non-drinkers to form a dichotomous indicator for drinking status.

#### Smoking status

Smoking status was ascertained by asking respondents on how many days they smoked during the past month using the following response options: Yes, smoked every day; Yes, smoked on one or more days, but not every day; No days. Participants who smoked every day were classified as daily smokers while those who smoked on one or more days, but not every day were classified as occasional smokers. Current non-smokers were defined as those who did not smoke in the past month, including never-smoker and former smoker [32–34]. Categories for occasional smokers and daily smokers were combined and compared to non-smokers to form a dichotomous indicator for smoking status [22].

#### Gifting alcohol behavior

Gifting alcohol included two types of behaviors, offering and receiving alcohol. Offering alcohol was defined as offering at least one unopened bottle of alcohol as a gift to others in the past year. Receiving alcohol was defined as receiving at least one unopened bottle of alcohol as a gift from others in the past year.

### Data analysis

The data was exported from the survey platform to Microsoft Excel and then uploaded to SPSS (version 22.0) for statistical analysis. Descriptive statistics for socio-demographic characteristics, social capital, and alcohol gifting behaviors are reported. The significance of differences between offering and receiving alcohol gifts across socio-demographic characteristics was determined using Chi-square analyses. Differences that reached statistically significance were included in a multiple logistic regression. The significance of each coefficient in the model was determined using the Wald test. Adjusted odds ratios (AORs) were used to express the odds of offering/receiving alcohol compared to the odds of not offering/receiving alcohol for each covariate, controlling for other covariates in the model. To determine the association between gifting alcohol and alcohol use and cigarette use, six logistic regression models were constructed where substance use was included as the outcome. The demographic characteristics which were significantly associated with smoking and drinking in the univariate analysis were included as covariates in the six multiple logistic regression models. Model 1 assessed the relationship between offering gifted alcohol and alcohol use. Model 2 assessed the relationship between receiving gifted alcohol and alcohol use. Models 3 included both offering and receiving gifted alcohol as covariates, with alcohol use as the outcome. Model 4 assessed the relationship between offering gifted alcohol and tobacco use. Model 5 evaluated the relationship between receiving gifted alcohol and tobacco use. Model 6 included both offering and receiving gifted alcohol with tobacco use as the outcome.

## Results

### Individual sociodemographic characteristics and drinking behavior

The average age of the participants was 47.8 (SD 9.3) years old, with 39.2% of the participants aged 45–49 years. Participants were majority male (82.5%) and married (88.2%). More sociodemographic characteristics were shown in Table 1. Almost half of the participants reported being current drinkers, of which 6.2% were daily drinkers and 38.4% were occasional drinkers.

**Table 1:** The distribution of alcohol gifting

**Variables**	**N (%)**	**Gifting alcohol**			
		**Offering** **n (%)**	***P***	**Receiving** **n (%)**	***P***
**Total**		452(29.9)		656(43.4)	
**Age**			0.001**		0.002**
< 45	336(22.2)	96(28.6)		119(35.4)	
45–49	593(39.2)	208(35.1)		281(47.4)	
50 +	583(38.6)	148(25.4)		256(43.9)	
**Gender**			0.002**		< 0.001**
Male	1248(82.5)	394(31.6)		582(46.6)	
Female	264(17.5)	58(22.0)		74(28.0)	
**Ethnicity**			0.994		0.391
Han	1502(99.3)	449(29.9)		653(43.5)	
Minority	10(0.7)	3(30.0)		3(30.0)	
**Marital status**			< 0.001**		< 0.001**
Married	1334(88.2)	420(31.5)		606(45.4)	
Others	178(11.8)	32(18.0)		50(28.1)	
**Place of residence**			0.005**		0.849
Rural area	715(47.3)	185(25.9)		315(44.1)	
Micropolis	437(28.9)	143(32.7)		185(42.3)	
Large- and-medium size cities	360(23.8)	124(34.4)		156(43.3)	
**Education**			0.026*		0.024*
Elementary school or less	282(18.7)	65(23.0)		109(38.7)	
Junior high school	595(39.4)	182(30.6)		285(47.9)	
High school	353(23.3)	108(30.6)		151(42.8)	
Junior college, college or higher	282(18.7)	97(34.4)		111(39.4)	
**Household annual income (RMB)**			< 0.001**		0.968
< 20,000	494(32.7)	128(25.9)		198(40.1)	
20,000–49,999	479(31.7)	128(26.7)		204(42.6)	
50,000–79,999	208(13.8)	60(28.8)		83(39.9)	
80,000–99,999	122(8.1)	45(36.9)		63(51.6)	
100,000 +	209(13.8)	91(43.5)		108(51.7)	
**Province**			< 0.001**		0.016*
Guangdong	982(64.9)	238(24.2)		404(41.1)	
Shaanxi	530(35.1)	214(40.4)		252(47.5)	
**Smoking status**			0.159		< 0.001**
Daily smoker	501(33.1)	155(30.9)		250(49.9)	
Occasional smoker	133(8.8)	48(36.1)		71(53.4)	
Nonsmoker	878(58.1)	249(28.4)		335(38.2)	
**Drinking status**			< 0.001**		< 0.001**
Daily drinker	93(6.2)	36(38.7)		62(66.7)	
Occasional drinker	580(38.4)	255(44.0)		327(56.4)	
Nondrinker	839(55.5)	161(19.2)		267(31.8)	
**Cognitive social capital**			0.205		0.012*
High score	705(46.6)	222(31.5)		330(46.8)	
Low score	807(53.4)	230(28.5)		326(40.4)	
**Social network**			0.004**		0.002**
High score	644(42.6)	218(33.9)		309(48.0)	
Low score	868(57.4)	234(27.0)		347(40.0)	
**Social participation**			< 0.001**		< 0.001**
High score	696(46.0)	262(37.6)		337(48.4)	
Low score	816(54.0)	190(23.3)		319(39.1)	

### The correlates of alcohol gifting

The study showed that 43.5% of participants had received alcohol, and 29.9% had offered alcohol. The results from the Chi-square tests in Table 1 demonstrated that age, gender, marital status, education, region, social network, and social participation were all significantly associated with both offering and receiving alcohol. Place of residence and household annual income were only associated with offering alcohol, while smoking status and cognitive social capital were only associated with receiving alcohol. Ethnicity was unrelated to either offering or receiving alcohol.

The results from the multiple logistic regression analysis in Table 2 further showed that participants aged 45 to 49 years old were more likely to offer alcohol than the people aged more than 50 years old (AOR = 1.4; 95% CI: 1.07–1.83). Meanwhile, those younger than 45 years were less likely to receive alcohol (AOR = 0.71; 95% CI: 0.52–0.98) than those older than 50 years. The male household heads were 2.34 times (95% CI: 1.51–3.61) more likely to offer alcohol and 1.40 times (95% CI: 1.02–1.93) more likely to receive alcohol than the female household heads. Participants from Shaanxi Province had higher odds of offering alcohol (AOR = 2.32; 95% CI: 1.81–2.97) and receiving alcohol (AOR = 1.42; 95% CI: 1.12–1.81) than participants from Guangdong Province. Drinking status and social participation were also significantly associated with offering and receiving alcohol. Participants who were daily drinkers (AOR offering = 2.69, AOR receiving = 4.01) and occasional drinkers (AOR offering = 3.05, AOR receiving = 3.91), or had a higher frequency of social participation (AOR offering = 1.84, AOR receiving = 1.37), were more likely to both offer and receive alcohol as a gift. We also observed that participants whose annual household income was more than one hundred thousand yuan were more likely to offer (AOR = 1.9; 95% CI: 1.30–2.78) and receive (AOR = 1.61; 95% CI: 1.11–2.36) alcohol than those with annual household income of less than 20,000. Similarly, those whose annual household income was between 80,000 and 100,000 yuan were more likely to receive alcohol (AOR = 1.81; 95% CI: 1.17–2.83). In addition, we observed that married participants (AOR = 1.62; 95% CI: 1.10–2.38), participants with an education level of junior high school (AOR = 1.61; 95% CI: 1.14–2.27), and participants with a large social network (AOR = 1.27; 95% CI: 1.01–1.58) had higher odds of receiving alcohol compared to those who were not married, had education level of junior college, college or higher, and had a small social network.

**Table 2:** Logistic regression results of factors associated with gifting alcohol

	**Gifting alcohol** **Odds Ratio (95%C. I.)**	
	**Offering Model**	**Receiving Model**
**Age**		
< 45	1.02(0.72–1.43)	0.71(0.52–0.98)*
45–49	1.40(1.07–1.83)*	1.08(0.84–1.38)
50 +	1.00	1.00
**Gender**		
Male	2.34(1.51–3.61)**	1.40(1.02–1.93)*
Female	1.00	1.00
**Marital status**		
Married		1.62(1.10–2.38)**
Others		1.00
**Education**		
Elementary school or less		1.21(0.81–1.80)
Junior high school		1.61(1.14–2.27)**
High school		1.15(0.80–1.636
Junior college, college or higher		1.00
**Household annual income (RMB)**		
< 20,000	1.00	1.00
20,000–49,999	0.81(0.60–1.11)	0.95(0.72–1.25)
50,000–79,999	1.01(0.68–1.48)	0.98(0.68–1.40)
80,000–99,999	1.58(0.99–2.51)	1.81(1.17–2.83)**
100,000 +	1.90(1.30–2.78)**	1.61(1.11–2.36)*
**Province**		
Guangdong	1.00	1.00
Shaanxi	2.32(1.81–2.97)**	1.42(1.12–1.81)**
**Drinking status**		
Daily drinker	2.69(1.68–4.32)**	4.01(2.51–6.41)**
Occasional drinker	3.05(2.38–3.91)**	2.47(1.97–3.11)**
Nondrinker	1.00	1.00
**Social network**		
High score		1.27(1.01–1.58)*
Low score		1.00
**Social participation**		
High score	1.84(1.43–2.36)**	1.37(1.09–1.73)**
Low score	1.00	1.00

^*^p < 0.05; **p < 0.01

### Association between gifting alcohol and drinking and smoking use status

The results from Table 3 demonstrated that receiving alcohol was associated with current alcohol use (AOR = 2.68; 95% CI: 2.16–3.34) and current tobacco use (AOR = 1.38; 95% CI: 1.10–1.72), while offering alcohol was only associated with current alcohol use (AOR = 3.06; 95% CI: 2.41–3.89), adjusting for sociodemographic characteristics and social participation. In addition, both alcohol offering and receiving were still significantly associated with drinking and smoking status even when controlling for the other gifting behavior. The household heads who offered (AOR = 2.16, 95% CI: 1.63–2.85) or received alcohol (AOR = 1.87, 95% CI: 1.45–2.41) had higher odds of being current drinkers than those who didn’t offer or receive alcohol. Additionally, those who received alcohol were more likely to be current smokers (AOR = 1.64; 95% CI: 1.25–2.14), while those who offered alcohol were less likely to be current smokers (AOR = 0.71; 95% CI: 0.53–0.95).

**Table 3:** Logistic regression analysis for drinking and smoking behaviors predicting by gifting alcohol

	**Current drinking status**			**Current smoking status**		
**Gifting alcohol**	**Model 1**	**Model 2**	**Model 3**	**Model 4**	**Model 5**	**Model 6**
**Offering**						
Yes	3.06(2.41–3.89)**		2.16(1.63–2.85)**	0.95(0.74–1.21)		0.71(0.53–0.95)*
No	1.00		1.00	1.00		1.00
**Receiving**						
Yes		2.68(2.16–3.34)**	1.87(1.45–2.41)**		1.38(1.10–1.72)**	1.64(1.25–2.14)**
No		1.00	1.00		1.00	1.00
**Gender**						
Male	3.74(2.67–5.24)**	3.45(2.47–4.83)**	3.50(2.50–4.91)**	17.19(9.86–29.96)**	16.29(9.34–28.40)**	16.43(9.41–28.67)**
Female	1.00	1.00	1.00	1.00	1.00	1.00
**Education**						
Elementary school or less				2.24(1.52–3.29)**	2.25(1.53–3.30)**	2.18(1.49–3.21)**
Junior high school				1.52(1.09–2.11)*	1.48(1.06–2.06)*	1.45(1.04–2.03)*
High school				1.29(0.91–1.85)	1.29(0.90–1.84)	1.27(0.89–1.82)
Junior college, college or higher				1.00	1.00	1.00
**Province**						
Guangdong				0.43(0.33–0.54)**	0.44(0.34–0.55)**	0.42(0.33–0.53)**
Shaanxi				1.00	1.00	1.00
**Social participation**						
High score	1.26(1.01–1.58)*	1.33(1.07–1.67)*	1.25(1.00–1.57)*			
Low score	1.00	1.00	1.00			

^*^p < 0.05; **p < 0.01

## Discussion

Research on alcohol has generally only focused on its use or overuse as a psychoactive substance [3, 35], meaning there are few studies on the behavior of alcohol gifting. In this study, two provinces in southern and northern China were selected to explore alcohol gifting, associated factors, and behavioral outcomes. We also distinguished between actively offering and passively receiving alcohol gifts. This study showed that nearly half of the participants had received alcohol, and nearly one-third had offered alcohol, suggesting that alcohol gifting is common in China.

There are some differences in alcohol gifting across sociodemographic characteristics. Our research showed that men were more likely to offer and receive alcohol than women. We posit two potential explanations for this difference. One, there are sex differences in drinking alcohol behavior in China, where drinking frequency in men is higher than in women [3, 35]. This higher drinking frequency in men might explain why alcohol gifting is more common among men. Two, compared to women, men are more likely to participate in social interaction where alcohol consumption is normative in the Chinese socio-cultural context, especially on business occasions.

In addition, the study also suggests that married people have higher odds of receiving alcohol. Consistent with this finding, a previous study of Chinese drinking behaviors showed that being married is associated with more alcohol consumption [36]. We hypothesize that married people may be more invested in maintaining interpersonal relationships than those who aren’t married, especially on special holidays when alcohol gifting is common. Married people may have more social and family ties that are accompanied by gift-giving expectations, and therefore may be more likely to receive alcohol as a gift. Moreover, Chinese society emphasizes filial respect, and gift-giving is a way for the younger generation to show respect for the elder generation. As married people are generally more mature and have higher status within the family hierarchy, they may be more likely to receive gifts such as alcohol.

The finding that participants with a high level of social participation were more likely to give and receive alcohol is consistent with the role that alcohol plays in Chinese culture, where alcohol consumption is commonly involved in social interaction. Chinese people traditionally consider drinking an important tool of social contact and emotional expression. Alcohol often accompanies business meetings, social activities, weddings, funerals, holidays, and other special celebrations [37]. Gift giving can reduce uncertainty while producing positive emotions, social cohesion, and commitment [38]. Feelings of obligatory reciprocity often accompany gift-giving, even when altruistic motives are also present [39]. As consequence, those who have received alcohol may feel obligated to reciprocate after receiving an alcohol gift by offering the gift-giver help, strong emotional ties, etc. While social participation was found to be significantly associated with offering alcohol, we did not find a significant relationship with cognitive social capital and social network. Social Exchange Theory holds that all human behaviors are exchange behaviors, and gift-giving is also a social exchange behavior [40]. In other words, the cognitive perception of social capital and the extent of social network might not promote the occurrence of gifting behaviors. Perhaps gift-giving behavior can only be promoted through social engagement, where there is real interaction with people in the context of social participation.

The behaviors of offering and receiving alcohol were also related to family annual income. Higher annual household income indicates higher economic status. It was previously illustrated that individuals of higher economic status are more likely to offer expensive wines to demonstrate their prestige and high social standing [41]. Our results offer additional support for the relationship between higher SES and alcohol gifting.

This study found that compared with Guangdong, the southern coastal area in China, the phenomenon of alcohol offering and receiving is more common in Shaanxi, an inland city in northwest China. This may be partially explained by the regional differences in drinking prevalence. According to a study on regional differences in alcohol consumption in China, the prevalence of regular drinking in the northern region is higher than prevalence in the central-southern region [42]. It is possible that northerners perceive drinking as an effective way to cope with cold weather, and northern culture emphasizes hospitality with frequent gatherings and exhortation to drink [43]. In addition, Guangdong has higher economic and cultural development than Shaanxi due to the advantages of economic reform and being a coastal area which has more foreign trade activities with the outside. This higher level of economic development may be associated with receiving more information about the harms of drinking, leading to more concern about its effects on health and avoidance of alcohol [44]. This difference may also be related to the cultural differences of gifting between the North and the South.

Drinking status was also found to be strongly associated with giving and receiving alcohol. Behavioral Susceptibility Theory posits that behavior will gradually increase when that behavior is convenient [45]. Drinkers are more likely to approve of drinking than non-drinkers and may have more regular, convenient access to alcohol. Drinkers may therefore be more inclined to choose alcohol as gifts.

In another study in China, smoking outcomes were associated with cigarette gifting behaviors [22]. Notably, a similar relationship was found in the current study, where gifting alcohol was significantly associated with not only drinking, but also smoking. Many studies have demonstrated that tobacco and alcohol were complementary products, and co-use is common [46–49]. Drinkers are also more likely to smoke cigarettes than nondrinkers [50]. These findings may indicate that receiving alcohol as a gift may facilitate the consumption of addictive substances including tobacco and alcohol.

With regards to policy implications, the result of the current study can be used to inform prevention and intervention. First, alcohol gifting is associated with higher odds of current drinking and current smoking. Previous studies have suggested that limiting alcohol advertising is an effective intervention to control drinking [51, 52]. While it might be difficult to ban all alcohol advertising, restrictions could be pursued that restricts advertising from using gifting themes and imagery. Interventions that teach people how to refuse alcohol as gifts and suggest alternative gifts could also be pursued. Such interventions should be targeted at specific populations that have higher odds of alcohol gifting, for example, people who are male, married, currently drink alcohol, reside in the northern region, have larger social networks and more social participation, and people with higher economic status. Finally, given the differences in alcohol giving in North and South, local alcohol gifting culture should receive attention when formulating policies and interventions programs, particularly in the northern region.

### Limitations

Some limitations should be considered. First, the cross-sectional design prohibits causal associations. Additionally, self-reported questionnaires are vulnerable to recall bias and social desirability bias. Second, selection bias might misrepresent the prevalence of alcohol offering and receiving because the sample only included the household heads whose children were college students. Moreover, results may not generalize to the entire country, and the selected provinces might reflect north–south cultural differences due to their geographical and economic characteristics.

## Conclusion

In summary, the present study used a multistage sampling design to study alcohol gifting through both offering and receiving alcohol as a gift. The results showed that gender, household annual income, province, drinking status, social participation, and economic status were associated with alcohol offering and receiving. When investigating the relationship between alcohol gifting and alcohol and tobacco use, we found that alcohol receiving behaviors were significantly associated with both current alcohol and tobacco use.

## Data Availability

Because of the intellectual property policy of the funding body, the datasets generated and/or analyzed during the current study are not publicly available but are available from the corresponding author on reasonable request subject to approval from the funding body.
